# Growing a circular economy with fungal biotechnology: a white paper

**DOI:** 10.1186/s40694-020-00095-z

**Published:** 2020-04-02

**Authors:** Vera Meyer, Evelina Y. Basenko, J. Philipp Benz, Gerhard H. Braus, Mark X. Caddick, Michael Csukai, Ronald P. de Vries, Drew Endy, Jens C. Frisvad, Nina Gunde-Cimerman, Thomas Haarmann, Yitzhak Hadar, Kim Hansen, Robert I. Johnson, Nancy P. Keller, Nada Kraševec, Uffe H. Mortensen, Rolando Perez, Arthur F. J. Ram, Eric Record, Phil Ross, Volha Shapaval, Charlotte Steiniger, Hans van den Brink, Jolanda van Munster, Oded Yarden, Han A. B. Wösten

**Affiliations:** 1 Chair of Applied and Molecular Microbiology, Institute of Biotechnology, Technische Universität Berlin, Gustav-Meyer-Allee 25, 13355 Berlin, Germany; 2 Institute of Integrative Biology, University of Liverpool, Biosciences Building, Crown Street, Liverpool, UK; 3 TUM School of Life Sciences Weihenstephan, Technical University of Munich, Holzforschung München, Hans-Carl-von-Carlowitz-Platz 2, 85354 Freising, Germany; 4 Department of Molecular Microbiology & Genetics, Institute of Microbiology & Genetics, Georg-August-Universität Göttingen, Grisebachstr. 8, 37077 Göttingen, Germany; 5 Syngenta, Jealott’s Hill International Research Centre, Bracknell, Berkshire RG42 6EY UK; 6 Fungal Physiology, Westerdijk Fungal Biodiversity Institute & Fungal Molecular Physiology, Utrecht University Uppsalalaan 8, 3584 CT Utrecht, Netherlands; 7 Department of Bioengineering, Stanford University, 443 Via Ortega, Stanford, CA USA; 8 Department of Biotechnology and Biomedicine, Technical University of Denmark, 2800 Kongens Lyngby, Denmark; 9 Department Biology, Biotechnical Faculty, University of Ljubljana, Jamnikarjeva 101, 1000 Ljubljana, Slovenia; 10 AB Enzymes GmbH, Feldbergstr. 78, 64293 Darmstadt, Germany; 11 Department of Plant Pathology and Microbiology, Faculty of Agriculture, Food and Environment, The Hebrew University of Jerusalem, 76100 Rehovot, Israel; 12 Biotechnology Research, Production Strain Technology, Novozymes A/S, Krogshoejvej 36, 2880 Bagsvaerd, Denmark; 13 Quorn Foods, Station Road, Stokesley, North Yorkshire TS9 7AB UK; 14 Department of Medical Microbiology and Immunology, University of Wisconsin-Madison, Madison, 53706 USA; 15 Department of Molecular Biology and Nanobiotechnology, National Institute of Chemistry, Hajdrihova 19, SI-1000 Ljubljana, Slovenia; 16 Institute of Biology Leiden, Molecular Microbiology and Biotechnology, Leiden University, Sylviusweg 72, 2333 BE Leiden, The Netherlands; 17 French National Institute for Agriculture, Food and the Environment, INRAE, UMR1163, Biodiversité et Biotechnologie Fongiques, Aix-Marseille Université, Marseille, France; 18 MycoWorks, Inc, 669 Grand View Avenue, San Francisco, USA; 19 Faculty of Science and Technology, Norwegian University of Life Sciences, Droebakveien, 31 1430 Aas, Norway; 20 Chr. Hansen A/S, Bøge Alle 10-12, 2970 Hørsholm, Denmark; 21 The University of Manchester, Manchester Institute of Biotechnology (MIB) & School of Natural Sciences, 131 Princess Street, Manchester, M1 7DN UK; 22 Department of Biology, Microbiology, Utrecht University, Padualaan 8, 3584 CH Utrecht, The Netherlands

## Abstract

Fungi have the ability to transform organic materials into a rich and diverse set of useful products and provide distinct opportunities for tackling the urgent challenges before all humans. Fungal biotechnology can advance the transition from our petroleum-based economy into a bio-based circular economy and has the ability to sustainably produce resilient sources of food, feed, chemicals, fuels, textiles, and materials for construction, automotive and transportation industries, for furniture and beyond. Fungal biotechnology offers solutions for securing, stabilizing and enhancing the food supply for a growing human population, while simultaneously lowering greenhouse gas emissions. Fungal biotechnology has, thus, the potential to make a significant contribution to climate change mitigation and meeting the United Nation’s sustainable development goals through the rational improvement of new and established fungal cell factories. The White Paper presented here is the result of the 2nd Think Tank meeting held by the EUROFUNG consortium in Berlin in October 2019. This paper highlights discussions on current opportunities and research challenges in fungal biotechnology and aims to inform scientists, educators, the general public, industrial stakeholders and policymakers about the current fungal biotech revolution.

## Introduction

The term ‘biotechnology’ was coined by the Hungarian Karl Ereky [1] in 1919, the same year that Pfizer became the first company to commercialise a product manufactured by the controlled fermentation of a mould. The product Pfizer made was citric acid for commercial use as a flavouring agent, acidifier and chelating agent in food, beverages, and the pharma and chemical industries. It was produced biotechnologically with the mould *Aspergillus niger* [2], and 100 years later, citric acid is still produced with this filamentous fungus and has formed a fast growing multibillion Euro market for convenience foods and beverages [2, 3]. Many organic acids, enzymes, life-saving antibiotics and drugs are produced by filamentous fungi, and a lot of our foods and beverages would not exist at all without their fermentative capacities [4]. It is undisputed that filamentous fungal-based biotechnology is of pivotal importance to our daily lives. Many companies around the globe are leveraging the power of filamentous fungi, with major players in Europe that include: AB Enzymes, BASF, Bayer, Chr. Hansen, Dyadic International, DSM, DuPont, Kerry Group, Marlow Foods, Novozymes, Puratos, Syngenta and Roal Oy.

In a more recent endeavour, biologists, chemists, bioinformaticians, bioengineers, process engineers and material scientists have collaborated to turn by-products and waste from agriculture and forestry into composite materials with the help of mushroom-forming fungi. The vision offered by this group is disruptive: soon, we will sit on mushroom-created furniture, we will live in houses made from fungal hyphae, plastics and insulation materials in cars, trains, planes and spacecrafts will be composed of fungal composites and many of our textiles will be derived from fungi. In the not so distant future, new fungal-based products will be introduced into the market that offer similar or superior characteristics compared to classic petroleum-derived products, with a reduced or even negative carbon footprint and full biodegradability. Another vision is not so far-fetched at all: products made with animal leather will be superseded in quality and price by those made with pure fungal mycelium. Such replacements will have substantial implications for the livestock, textile and fashion industries.

Fungi growing as a yeast morphology have impacted society for millennia and have been instrumental for the production of bread, beer and wine. However, fungal species capable of the filamentous growth form, which are the focus of this White Paper, have additional beneficial properties such as the productions of a diverse array of metabolites, enzymes and materials. Indeed, Scientific American stated in 2019 that: “The mycelium revolution is upon us” [5]. A 100 years after the birth of fungal biotechnology, this platform technology is undergoing a renaissance by providing sustainable solutions for diverse industries and markets [6]. In the following, we will summarize current and anticipated products made from moulds and mushrooms and related new avenues of research. We will also highlight recommendations discussed during the EUROFUNG 2nd Think Tank consortium for ways to drive innovation in this renaissance through strategic and infrastructural measures for basic and applied science on filamentous fungi.

### The lifestyle of filamentous fungi—moulds and mushrooms

**Fig. 1:**
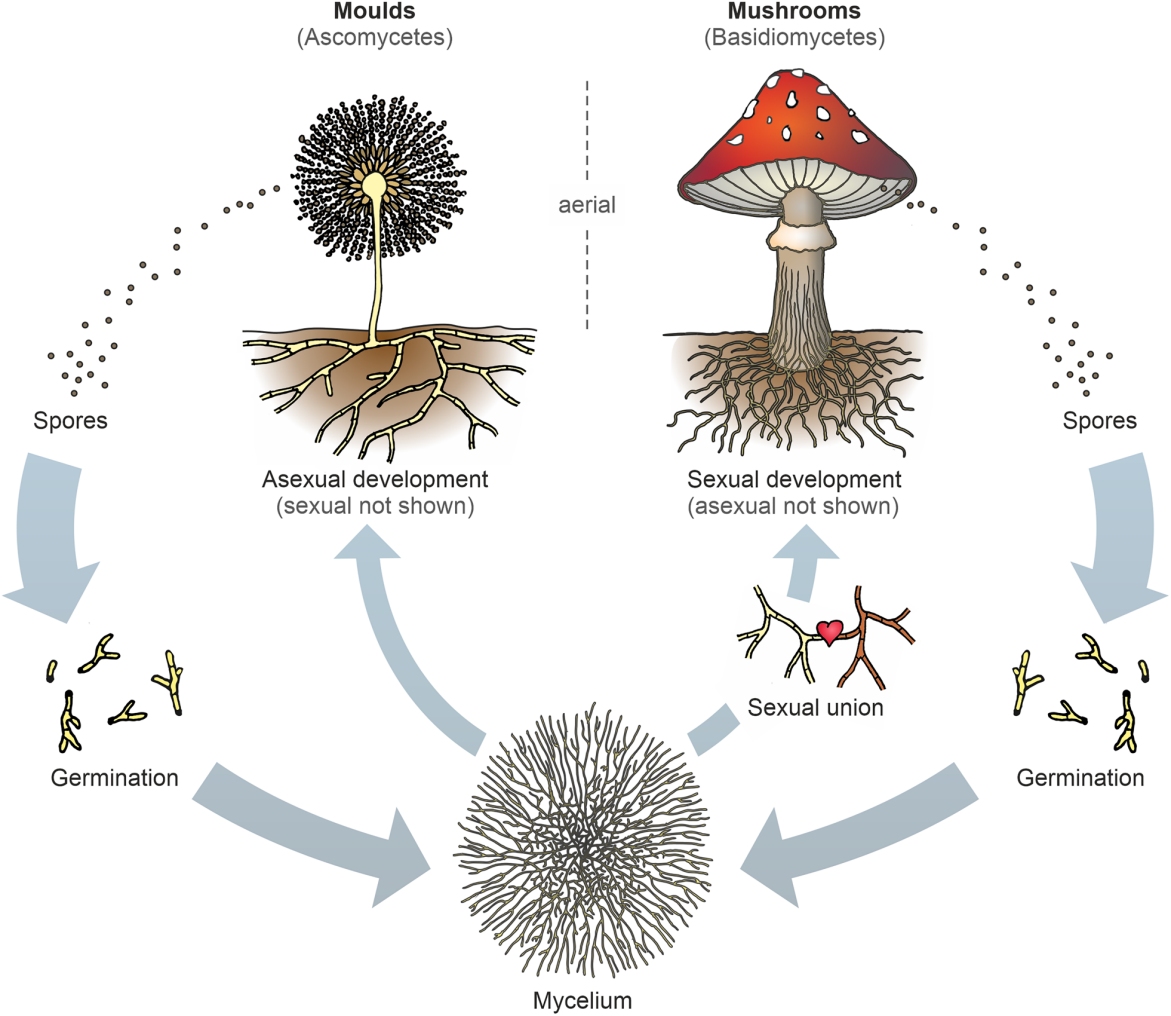
The fungal life cycle

The life of a filamentous fungus usually starts with a spore, which has a diameter of only a few microns (µm) (Fig. 1). The spore starts to swell in a humid and nutrient-rich environment and germinates. A germ tube is formed that elongates to eventually form a thread-like, filamentous cell, called a hypha. After the hypha grows and elongates for some time, it forms a network of interconnected hyphal threads called a mycelium. When nutrients become limited in the substrate within which the mycelium lives, the mycelium starts to explore the air and space in order to form reproductive structures. Ascomycetes (moulds) can form conidiophores that produce asexual spores at their ends and/or fruiting bodies that produce sexual spores inside them. Basidiomycetes are known for their ability to form fruiting bodies to generate sexual spores. Some of these fruiting bodies are colloquially called mushrooms. These fruiting bodies consist of mycelia which are more densely packed and different in their composition compared to the rather loose substrate mycelia, which forms three-dimensional net-like structures resembling the global system of interconnected computer networks.

Filamentous fungi are both invisible and visible. The diameter of fungal hyphae range from 2 to 10 µm, and a fungal mycelium consists of a network of mm- to cm-long hypha. The mycelia of mushroom-forming fungi can colonise large surface areas, as illustrated by an individual of the honey mushroom *Armillaria bulbosa* (common name honey mushroom in English and *Hallimasch* in German) which has colonised > 1000 hectares of forest soil, making it the largest and oldest organism on Earth [7]. Mycelia of mushroom-forming fungi can also be grown on various by-products and waste streams from forestry and agriculture. The efficiency of colonisation and biomass formation is determined by the composition and physical properties of the substrate, the environmental growth conditions (temperature, humidity and pH) and the genetic make-up of the fungus. A strain of *Schizophyllum commune* (common name split gill), for instance, in which two regulatory genes for mushroom formation are inactivated, produces threefold more biomass of fungal tissue when compared to the parental strain [8].

**Fig. 2:**
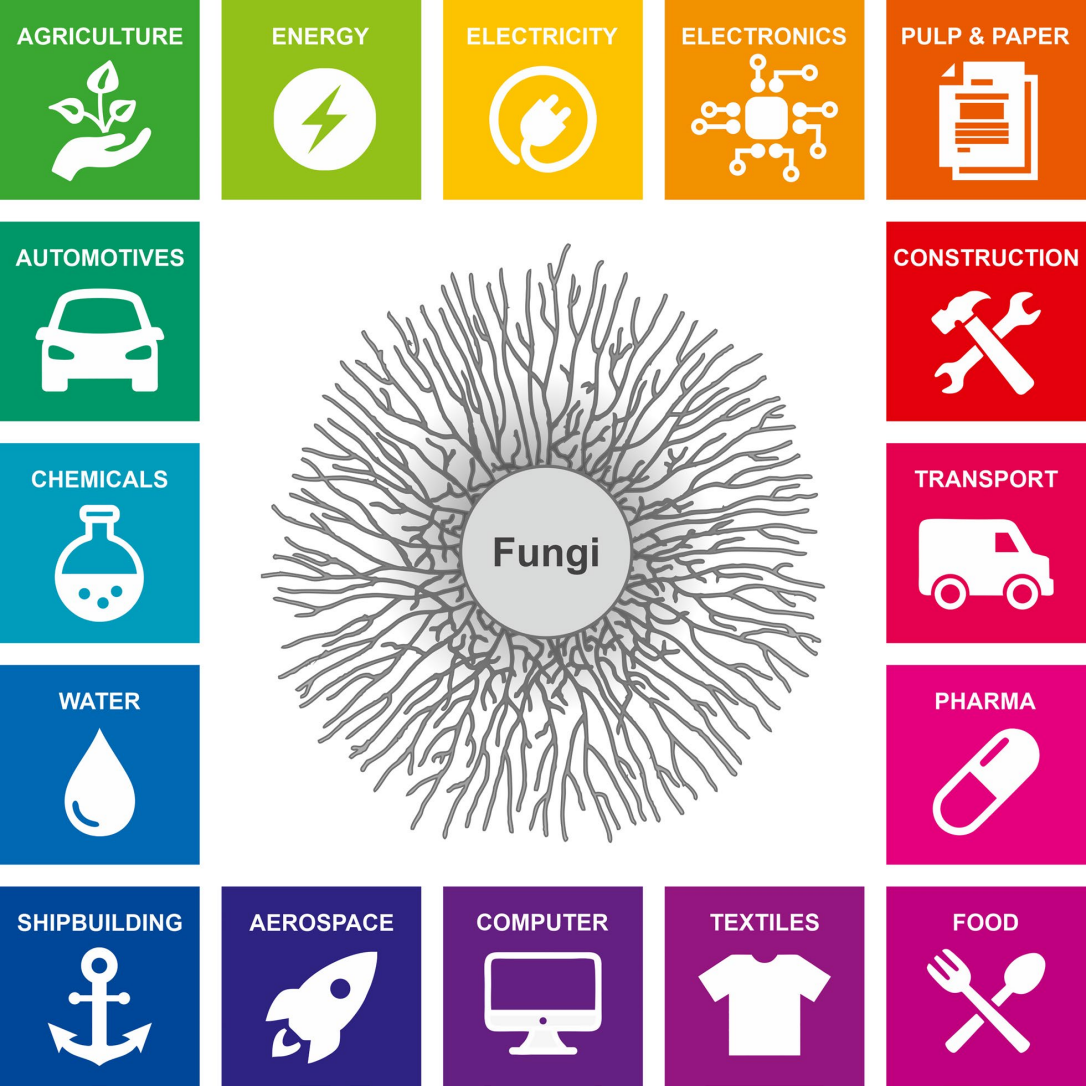
Industries profiting from the metabolic capacities of filamentous fungi

Filamentous fungi have evolved to become superbly efficient decomposers and have developed the ability to feed on and break down organic matter and polymeric substances [9]. Polysaccharides from plant biomass make up most of the biomass on Earth (450 out of a total of 550 gigatons of carbon; [10]) and represent the major carbon sources that drives fungal growth. In order to digest, humans must first ingest. Filamentous fungi first digest and then ingest. The fungal cells that infiltrate a substrate secrete enzymes, such as cellulases, amylases, pectinases, inulases, proteases and lipases, into the surrounding medium and hydrolyse (break down) plant polysaccharides (e.g. cellulose, starch, pectin, inulin), proteins and lipids. The degradation products of polysaccharides are monosaccharides, such as glucose, and oligosaccharides, which are subsequently taken up into the hyphae with the help of specific sugar transporters. This hydrolytic capacity for plant biomass, coupled with an extraordinary high secretion capacity for enzymes (up to 100 g/L are reported; [11]), forms the basis for the success of high-performance enzyme-producing cell factories, such as *A. niger, A. oryzae, Trichoderma reesei* and *Thermothelomyces thermophilus* (all being ascomycetes). Their enzyme products are exploited by a diverse array of major industries, including food and feed, detergent, pulp and paper, fuel, pharmaceutical and chemical (Fig. 2; [4]).

Notably, the predicted gene set for plant polysaccharide-degrading enzymes in these fungi is between 100 and 250, whereas only 30 can be found in the baker’s yeast *Saccharomyces cerevisiae*. The success of *S. cerevisiae,* which cannot grow on plant polymeric substances [12], or the bacterium *Escherichia coli* as platform organisms to produce biofuel (ethanol), jet fuel (terpenoids), fine and commodity chemicals is only guaranteed by the filamentous fungal cell factories mentioned above that provide *S. cerevisiae* and *E. coli* with simple carbon sources during the fermentation process. Hence, filamentous fungal cell factories play a central role in the sustainable production of biofuels and chemicals.

### The product portfolio of filamentous fungi—an overview

The product range of filamentous fungi is not limited to citric acid and enzymes (Table 1). In fact, the natural metabolic capacities of filamentous fungi are extraordinarily diverse and unmatched in nature. Organic acids, chemicals, antibiotics and other drugs, proteins and enzymes, meat alternatives, vitamins, polyunsaturated fatty acids and even composite materials and vegan leathers are existing fungal products. Table 2 highlights some currently traded pharmaceuticals derived from filamentous fungi.

**Table 1:** Selected list of established filamentous fungal cell factories and their products. (Modified after [13])

Filamentous fungus	Important Product(s)
*Acremonium chrysogenum*	β-lactam antibiotics (cephalosporins)
*Aspergillus niger*	Enzymes (glucoamylase, proteases, phytases, glucose oxidase) Organic acids (citric acid, gluconic acid)
*Aspergillus oryzae*	Enzymes (amylases)
*Aspergillus terreus*	Enzymes (xylanases) Organic acids (itaconic acid) Secondary metabolites (lovastatin)
*Blakeslea trispora*	Vitamins (β-carotene)
*Fusarium venenatum*	Mycoprotein as meat alternative
*Ganoderma lucidum*	Composite materials (packaging material, construction material) Imitation leather
*Mortierella alpina*	Polyunsaturated fatty acids used as food additives
*Mucor circinelloides*	Polyunsaturated fatty acids used as food additives
*Penicillium brevicompactum*	Mycophenolic acid
*Penicillium camemberti*	Cheese production
*Penicillium chrysogenum*	β-lactam antibiotics (penicillins) Enzymes (glucose oxidase)
*Penicillium nalgiovense*	Mould-fermented salami
*Penicillium roqueforti*	Cheese production
*Penicillium solitum*	Mevastatin
*Pleurotus ostreatus*	Food Composite materials (packaging material, construction material)
*Rhizopus oligosporus*	Tempeh production
*Thermothelomyces thermophilus*	Enzymes (cellulases, phytases, laccases)
*Trichoderma reesei*	Enzymes (cellulases, hemicellulases)
*Umbelopsis isabellina*	Polyunsaturated fatty acids used as biodiesel

**Table 2:** Selected pharmaceuticals derived from filamentous fungi and their applications. (Modified after [13])

Pharmaceutical	Remark
β-lactams	Penicillins and cephalosporins account for more than 30% of the global antibiotics market
Cyclosporin	Immunosuppressant that avoids organ rejection in transplant surgery
Drospirenone	Steroid hormone used as a birth control pill and traded as Slynd; in combination with an oestrogen traded under the brand name Yasmin
Echinocandins	Caspofungin, micafungin and anidulafungin used for the treatment of *Candida* infections
Griseofulvin	Antifungal used for the treatment of skin infections
Mycophenolic acid	Immunosuppressant that avoids organ rejection in transplant surgery and is traded as CellCept
Myriocin	Chemical analogue thereof is used to treat multiple sclerosis; approved in 2018 as *Gilenya*
Psilocybin	Indolalkaloid currently being tested in phase II clinical trials for the treatment of major depressive disorders and is considered by the FDA as a breakthrough therapy [16]
Statins	Lovastatin, simvastatin and pravastatin are used to treat cardiovascular diseases by lowering cholesterol levels

Note that *T. thermophilus* was formerly named *Myceliophthora thermophila* and that the *P. chrysogenum* strain used for penicillin production was recently reidentified as *P. rubens* [14, 15]. While this review focuses on the Dikarya lineage, that is the ascomycetes and basidiomycetes, five species in this list are found in another lineage, the Mucoromycota.

Some examples of current developments with fungal cell factories that will help to consolidate bio-economies and human health are summarized as follows.

*Aspergillus niger* is currently being explored by academia as a potential producer of other organic acids beyond citric acid, such as itaconate and galactarate. Itaconate could replace petroleum-based polyacrylic acid, which is a precursor for the polymer industry (absorbent polymers, polyester resins, synthetic latex) and galactarate could replace the current petroleum-based polyethylene terephthalate (PET) used for plastic production [17, 18].

*A. oryzae*, used to produce Asian food and beverages for over a 1000 years, has recently gained interest as a producer of malate, which has multiple applications in the food (acidulant, flavour enhancer), chemical (polyester resins) and pharmaceutical (acidulant) industries [19]. While several microbial cell factories from bacterial, yeast or filamentous fungal origin have been genetically engineered during the last few years to produce malate, *A. oryzae* is among the strains with the greatest yields [20].

*A. terreus* is exploited as a cell factory to produce two molecules: the organic acid itaconate, which is of interest for the polymer industry [21], and lovastatin, which is a cholesterol-lowering drug. Lovastatin has been marketed under the tradename Mevacor since the late 1980 s [22]. It, furthermore, serves as a starter molecule for manufacturing semisynthetic statins, such as simvastatin (trade name Zocor), which is the second leading statin on the market [23]. Current research is focused on re-routing lovastatin biosynthesis to one of its biosynthetic intermediates, monacolin J, which is the preferred precursor for Zocor synthesis [24]. Notably, a genetically engineered *A. oryzae* strain can reach monacolin J concentrations of about 5.5 g/L, which is considerably higher than has been achieved in heterologous hosts, such as *S. cerevisiae* (75 mg/L) or *Pichia pastoris* (renamed *Komagataella phaffii*; 600 mg/L) [25].

*P. chrysogenum* (*P. rubens*) is an antibiotics producer and the main cell factory used to produce penicillin and semisynthetic derivatives, with production levels up to 55 g/L [26]. Notably, the biosynthetic route for penicillin has recently been genetically reprogrammed towards pravastatin, which constitutes an interesting alternative to Zocor for the pharma industry [27].

*T. reesei* and *T. thermophilus* (formally *Myceliophthora thermophila*) are of commercial interest because of their ability to secrete cellulases and hemicellulases. These enzymes are key to converting lignocellulosic biomass into biofuel. Lignocellulose is composed of cellulose, hemicellulose and lignin and is a by-product of agriculture (e.g. straw, bagasse, corn stover) and forestry (e.g. sawdust). The thermostability of (hemi)cellulases from *T. thermophilus* are of special interest for current lignocellulose degradation processes, as high process temperatures are preferred in biorefineries to reduce the viscosity and increase the solubility of lignocellulosic biomass [28]. Genetically optimised hypersecreting strains have been established during the last two decades which achieve cellulase titres of 100 g/L [11]. These are the highest titres ever reported for protein secretion and exceed by 10–10,000-fold what can be achieved nowadays with bacterial, yeast or mammalian cell factories. Protein secretion titres in these cell factories are usually in the order of mg/L to only a few g/L [29–31]. Notably, *T. thermophilus* has been genetically reprogrammed to generate platform chemicals, such as fumarate, directly from renewable feedstocks to replace petroleum-based processes, which is of interest for the manufacturing of synthetic resins, biodegradable polymers (up to 17 g/L) and malate (up to 200 g/L) [32, 33]. The fungus has also been optimised by Dyadic International as a producer for biologics, including vaccines, therapeutic enzymes, proteins and biosimilars [34].

### Filamentous fungi as meat replacements

**Fig. 3:**
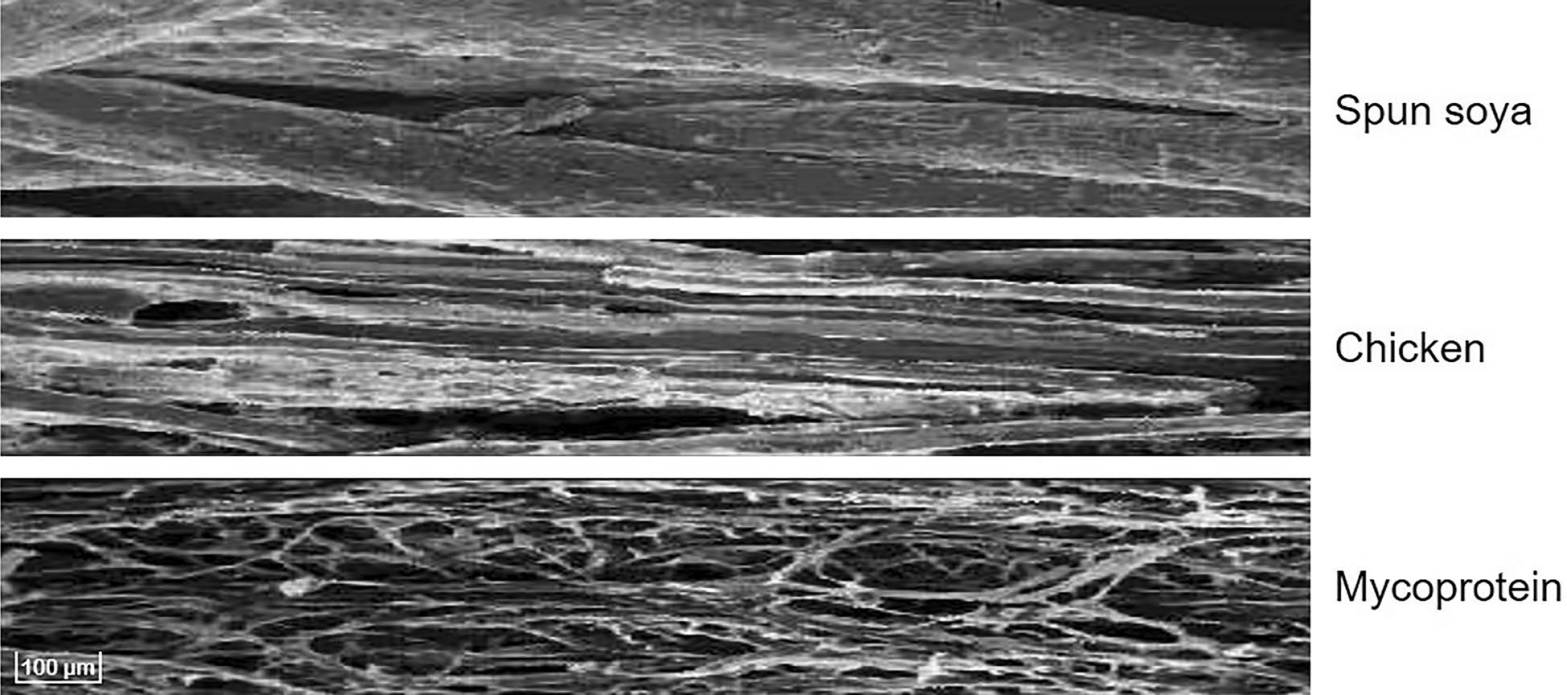
Unique textural attributes of Quorn™. Electron microscopic images of protein fibres from spun soya and chicken and hyphal filaments of *F. venenatum*. Bar, 100 µm

The use of fungi as a source of food predates recorded history [35]. These have been predominantly the mushrooms from supermarket shelves and foraging expeditions. The fruiting bodies of these fungi have a wide variety of tastes and textures and some of these are considered very meat-like, for example “Chicken of the Woods” (*Laetiporus sulphurous*) and “Beefsteak fungus” (*Fistulina hepatica*). There has recently been a move to create meat-like products from fungal mycelium grown in fermenters, rather than the solid fruiting bodies. This has allowed the introduction of ascomycetes, traditionally used as flavour modifiers in such foods as blue cheese, to enter the food chain as convincing meat substitutes. The longest established of these companies is Marlow Foods, using *Fusarium venenatum* under the trade name Quorn™, but several other companies have recently shown an interest in this area, including Mycorena (*A. oryzae*; [36]), Sustainable Bioproducts (*F. oxysporum*; [37]) and MycoTechnology, using the basidiomycete *Lentinula edodes* [38]. The hyphae of filamentous fungi, when aligned and organised, provide a structure that looks like and has the mouth feel of meat, particularly chicken, due to the similarity of fibre size (Fig. 3). The high amino acid and fibre content, and low saturated fat, combined with the high digestibility of fungal protein (see Additional file 1: Table S1), make this an exceptionally healthy food [39].

**Fig. 4:**
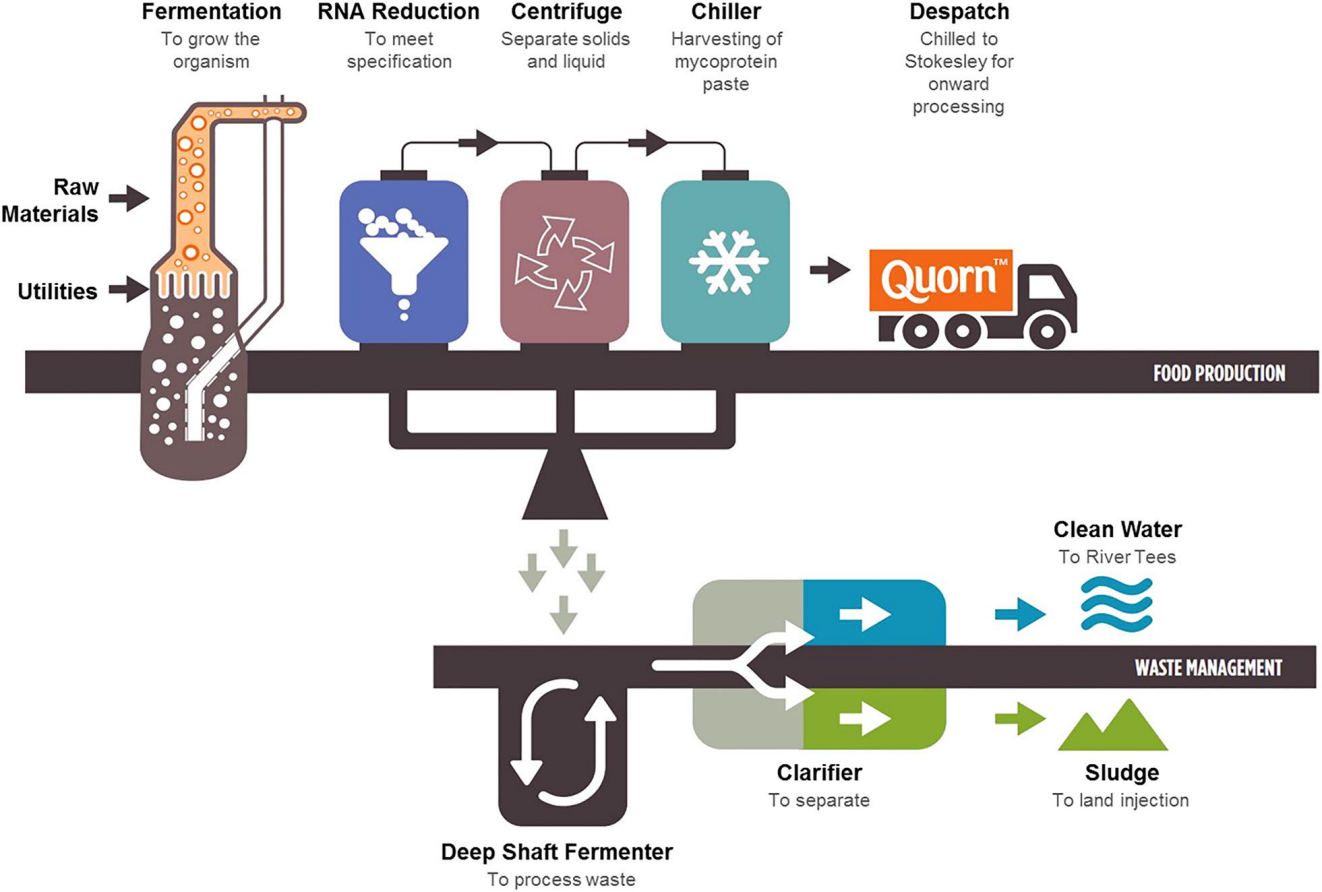
The Quorn™ fermentation process. A continuous supply of medium is fed into the fermenter and the broth is harvested simultaneously. The harvested broth is heated to a temperature that destroys proteases but leaves RNAses active, allowing the RNA content of the mycelium to be reduced to less than 2%, which is a regulatory requirement. Once the broth has been heat-treated, the mycelium is spun down to form a paste, which is mixed with binders and flavouring agents before being shaped, cooked and frozen. The supernatant from the paste is currently sent for treatment as wastewater, but active research at Marlow Foods is looking into how the 1.5% solids in the waste can be recovered as a food grade co-product

The fungi for food replacements are grown in fermenters on simple salts and glucose as the carbon source. Quorn™ uses air lift pressure cycle fermenters (Fig. 4). These are 30 m tall and have an operating volume of approximately 150 m^3^, which offer several operational and control benefits over conventional fermenters, including low shear stress allowing longer (and, therefore, higher quality) hyphae.

Glucose can be provided from several sources, currently by enzymatic digestion of starch from wheat or maize using the enzyme glucoamylase from *A. niger*, but lignocellulose is being considered for the future. As the protein from the original grain is retained for other uses and not fed to *F. venenatum*, the metric of the amount of protein per hectare, used to compare many animal proteins, is misleading when applied to fungi. The use of protein in animal feeds, whether animals or insects, acts to concentrate (with losses) what is present in the original, whereas fungi add protein to a protein-free feedstock. Furthermore, production of fungal protein is highly water-efficient in comparison to animal protein, requiring about one-tenth that of beef and half that of chicken. Quorn Foods was the first global meat alternative brand to achieve third party certification of its carbon footprint figures [40]. However, there is considerable potential to reduce the ecological impact of fermented *F. venenatum* further, and, as such, Marlow Foods are pursuing a research programme involving multiple external partners looking at water reduction, carbohydrate choice, fermenter optimisation, co-product valorisation and more.

### Filamentous fungi as biomaterials

Fungi thrive by decomposing and consuming dead plants by breaking down the plant’s cellulose, lignin and other sugars, then rearranging these molecules into their own biomass to grow. Their cell wall is secured by chains of chitin and glucans, which, like cellulose and keratin, are naturally forming polymers found in the toughest of organic tissues. Chitin is the same ingredient that creates the durable and flexible exoskeletons of insects and shellfish [41]. During the colonisation of substrates, hyphae bind the organic particles together, while degrading them simultaneously. A composite material is obtained, consisting of a bulk of organic substrate bound together by the hyphal network, by inactivating the fungus before the substrate is degraded (e.g. by drying or by heat inactivation). Pure fungal materials are obtained by complete degradation of the substrate or removing the fungal skin from the substrate. Both pure and composite mycelium can be used for different applications [42].

**Fig. 5:**
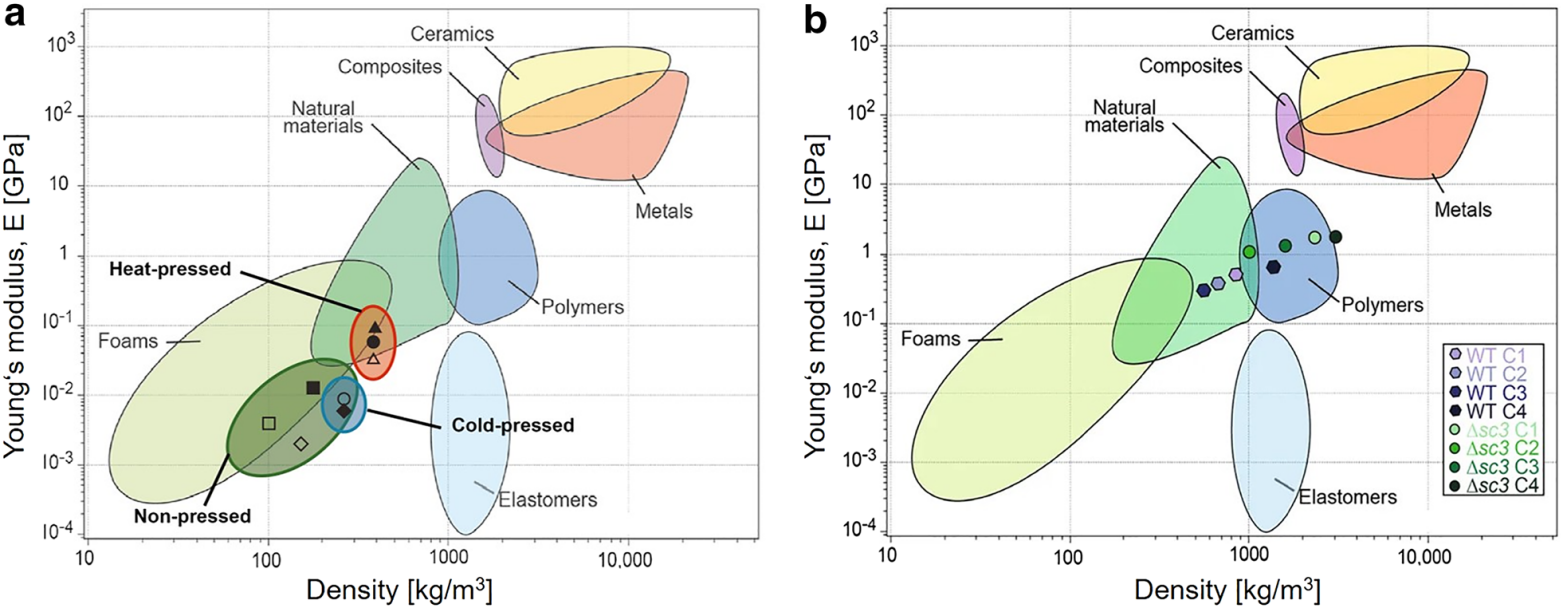
Material properties that can be achieved with fungal mycelia. **a** Mycelium composites. **b** Mycelium textiles. The pictures depicted are reproduced from [45], which has been published under a Creative Commons Attribution licence (CC BY, http://creativecommons.org/licenses/by/4.0/)

Stopping fungal growth during colonisation of the substrate results in materials with properties similar to that of expanded polystyrene or other foams (Fig. 5; [43, 44]). Such materials can be used as packaging material or for heat or acoustic insulation. The properties of both composite and pure mycelium materials are dependent on the substrate, the type of fungus, the growth conditions and post-processing. Heat pressing, for instance, improves the homogeneity, strength and stiffness of the mycelium composite material, shifting its performance from foam-like to cork- and wood-like (Fig. 5; [45]). A range of mycelium composite materials with different properties can be produced [46]. Future studies should focus on the coating of mycelium materials to reduce water uptake and volatile organic compound escape. The genetic engineering of strains, however, may make coating superfluous.

Mushroom-based materials have the potential for uses in place of leather, textiles and some plastics. The strength of pure mycelium of a wild-type *S. commune* strain is similar to that of natural materials, such as wood and leather [47]. By contrast, a material can be obtained by deleting a gene that encodes a protein called hydrophobin, that pearls off water droplets from fruiting bodies, with a strength similar to that of thermoplastics. The difference in the material properties between these strains is explained by a better packing of the hyphae in the deletion strain, resulting in an increased density of the mycelium. In addition, the absence of the hydrophobin in this strain results in a mycelium that is no longer water-repellent, consequently, it absorbs more water and dries more slowly than the wild-type [47]. Thus, mycelium-based materials can provide desirable mechanical properties which can also be bio-performative, for example, by buffering water availability. Future studies, therefore, should focus on not only the mechanical but also bioactive properties, making mycelium a potential source of new innovative materials with advanced properties. Future studies should also reveal whether mycelia can be utilised to develop materials with properties similar to the material families of the elastomers, composites, metals and ceramics (Fig. 5).

**Fig. 6:**
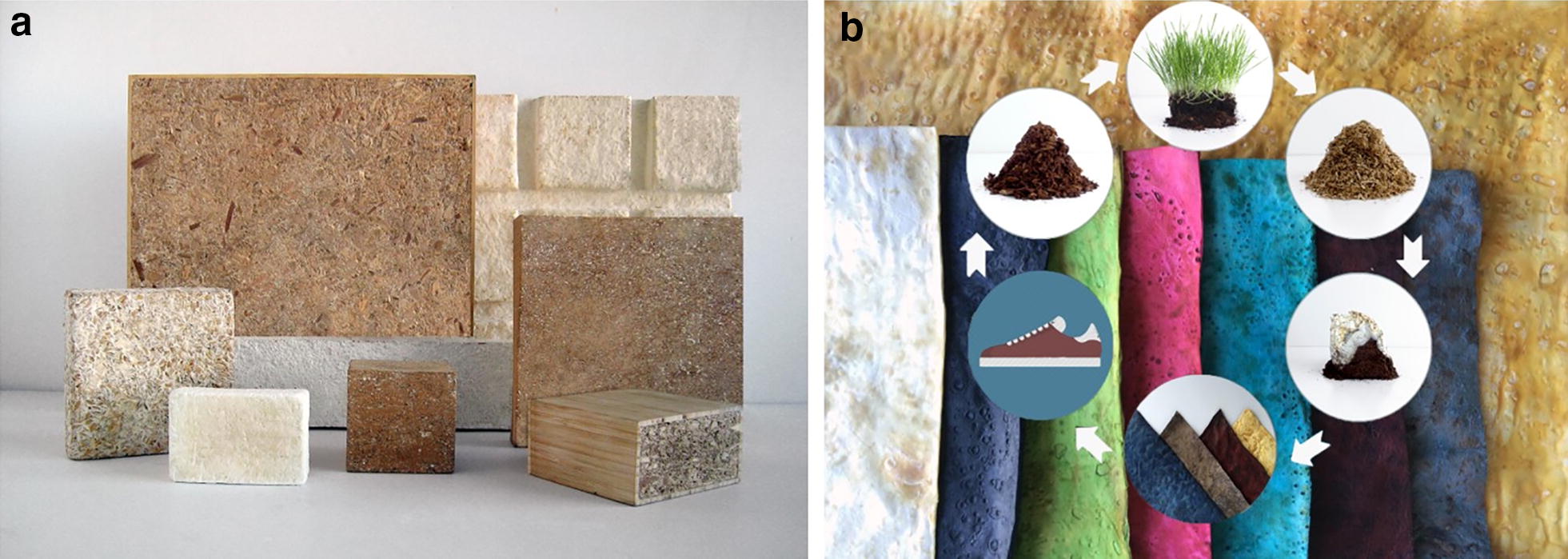
MycoWorks’ fungal analogues for composites and leather. **a** Analogues for synthetic wood composites and expanded polystyrene foams. Mushrooms are very sensitive to their surroundings, and it is possible by altering subtle factors to make their tissue express a range of variably determined physical characteristics. While these materials can be grown into building components for construction and interior architectures, they can also be grown with delicately tuneable qualities. The strength, durability and biodegradable nature of mushroom-based materials suggest many ways in which fungi may be used. When the material is processed with traditional industrial wrapping and laminating equipment, it is possible to create functional materials. **b** Analogues for animal leather. The MycoWorks technology is able to tune fine mycelium leather to have material advantages similar to animal skin, becoming supple, elastic and strong, with excellent return, drape, compression and insulation. This mycelium leather, launched in early 2020 as Reishi™, has been designed as a drop-in material for existing leather processing machine tools, where it can be cured, finished and manufactured using well-honed industrial techniques and formulas

The flexibility and aesthetics of mushroom materials were first appreciated by artists and designers who used them to grow living art works [48, 49]. Similar to cement and plaster, mycelia will bind, harden and set into a variety of solidified configurations. These designers and artists soon discovered that materials grown from this dense living matrix could be used to make advanced composites, foams and performance plastics, and the commercial potential soon followed. Companies that are designing and engineering mycelium materials include MycoWorks [50], Ecovative Design [51], NEFFA [52] and MOGU [53], to name but a few. Research programmes at MycoWorks proved that fine mycelium materials can be grown into the texture of sheet urethanes, animal skins and foams, with surfaces that are velvety and fluffy, leathery and rubbery, or beetle-shell brittle and shiny. After a mycelium-based object has been grown, it can be cut, processed and machined like many other materials. Demand for these materials has been driven by concerns for stabilizing the price of supply chains into the future. One of the first markets for which fungal mycelium can provide a solution is the fashion industry, where there is both a consumer and market demand for alternatives to the animal skins and plastics used to make apparel. A sensual and strong material for luxury fashion has been developed by moulding the body of the mycelium into a sheet and processing it in many of the same ways as animal leather (Fig. 6).

Notably, mushroom-based materials can absorb and dissipate a variety of energetic forces, ranging from sounds to seismic waves to ballistics [54]. They are naturally flame retardant, good thermal insulators and can be grown as flexible or rigid as one desires [50, 55]. While incredibly strong and durable, these materials can readily be broken down into constituent minerals and dispersed easily back into the world. Materials grown through a process of fermentation and decomposition require far less energy, water and other resources than conventional manufacturing.

### Filamentous fungi and a wood-based bio-economy

In order to shift away from a fossil-based economy and mitigate climate change triggered by the continuous industrial overproduction of CO_2_, many countries around the world, including those of the European Union, support the transformation to a renewable, bio-based and resource-efficient economy. Wood is considered to play a key role in the efforts towards this goal, due to the large potential of forests and wood products to sequester carbon [56, 57]. The term “sustainable” originates from forestry, initially coined by a German mining administrator, Hans Carl von Carlowitz, more than 300 years ago, who oversaw the timber supply of silver mines and realised that regrowth did not match the consumption rates [58]. Although the concept of “sustainability” has substantially broadened over time, it became a guiding concept in forestry, and recent surveys are demonstrating that European forests are still showing positive net annual increments [59] and are, therefore, actively storing carbon—a prerequisite for any economy that strives to become net carbon-neutral.

Current wood utilisation includes three major streams: energy, wood products, and pulp and paper, with about 60% going towards materials and 40% into energy in Europe. In this way, about 20% of the total carbon in use is annually sequestered into long-life materials [60]. However, in order to keep carbon stored within materials for as long as possible, a circular economy will be beneficial, including both recycling and reuse of wood products in a cascading fashion [61] before a final utilisation for energy services. Since an increased demand for and scarcity of wood can be expected with the transition to a bio-based economy, such resource efficiency will become even more important in the future. While high-quality wood would, therefore, be optimally used as timber for solid products initially, later utilisation steps in a virtual cascade would focus on the inherent fibres, polymers and monomeric wood constituents, such as sugars and lignans. This is where filamentous fungi have their highest potential to assist due to their natural ability of selectively and efficiently degrading and/or modifying all of these major wood constituents.

Due to the natural ability of fungi as heterotrophic organisms to efficiently degrade lignocellulosic plant biomass and convert the constituent sugars into energy-rich molecules, filamentous fungi or fungal enzyme cocktails have a high potential to be employed in the upgrading of wood in biorefinery applications towards second-generation biofuels or chemicals [62]. Up to now, however, mostly straws are used as substrate for these processes (e.g. the Sunliquid^®^ process by Clariant), with only a few examples of wood conversion (e.g. bioethanol from Borregaard); wood being much more recalcitrant, due to generally higher lignin contents among other things [63]. Considering all of the above, an integration of biotechnological processes using fungal platforms into pulp and paper mills which are already performing (chemical-based) wood separation, such as Kraft pulping, can be envisioned as having substantial technological and economic advantages [64].

Such “wood-based biorefineries” already exist—mostly in Scandinavia and eastern Europe [65]. Nevertheless, most of these are far from being true multi-product factories. Optimally, biorefineries would integrate biomass conversion processes and equipment to produce bioenergy and a range of products, including pulp, bio-based chemicals (e.g. for food and pharma), biofuels, electricity and heat. Regarding efficiency, the ability to use multiple feedstocks, such as those originating from pulping processes (pulpwood, extracts from effluents, fractions of pulping liquors), by-products from sawmills (e.g. wood shavings), or recovered materials (e.g. recycled paper and wood/fibre waste), would be advantageous. Many of these substrates are unsuitable for chemical upgrading due to their low purities and would have to undergo costly cleaning steps. Filamentous fungi, on the other hand, have the potential to circumvent these problems, since many are surprisingly robust against inhibitors and can selectively transform desired target compounds out of complex mixtures, including lignin-derived molecules [66].

In summary, wood-based biorefineries can play a central role in the transition towards sustainable economies, with the ability to generate commercial resources with a smaller carbon footprint of resources and emissions than traditionally produced materials (such as concrete). However, which product systems are the most promising ones regarding sustainability and efficiency must be assessed. Filamentous fungi can be of great value in these efforts and help to move more wood from energy into material use and, thus, to higher value creations. Nevertheless, substantial challenges and knowledge gaps can be identified that need to be addressed to be successful in the mid- to long-term:

- Research in wood decomposition is lagging behind that for more readily degradable biomass, such as agricultural residues and straws.
- Considering the benefits of using recovered (pre-used) wood products, pulp mill side streams and forestry/wood industry residues, the research and adaptation of fungal cell factories should include these to a much greater extent than done today.
- Climate change is slowly altering the tree composition, and it can be expected that hardwoods, such as beech and oak, will replace the more drought- and pathogen-sensitive softwoods, such as the currently predominating spruce, in the future. These types of wood differ in their composition, with clear implications for fungal degradation [67, 68]. Directed or engineered fungal adaptation and the optimisation of fungal enzyme cocktails will be needed to address these differences in order to optimise utilisation.
- Lignin is the most underutilised of all wood polymers due to its complex structure and difficulties in processing. It forms an amorphous network of phenyl propane units coupled to hemicellulose via ester linkages. It is the second most abundant natural material on Earth after cellulose. The worldwide production of lignin is approximately 100 million tons annually [69]. One of the main challenges of lignin valorisation lies in its diverse structure and poor solubility. Most of the lignin obtained (Kraft lignin/black liquor) is incinerated directly in the pulping plants to recover energy. Only about 2% of the technical lignin globally available (~ 5 million tons) is currently utilised. Basidiomycetes are unique in their vast abilities to modify and/or mineralise lignin, and these organisms should play a central role in developing the utility of this highly promising polyphenol. The potential of lignin as a material reservoir will be achieved through much more rigorous characterisation of the structure/function predictability achieved through these fungal transformations.

### Filamentous fungi to mitigate plastic pollution

Plastics are widely used in the global economy because they are inexpensive, versatile, lightweight and durable. Millions of tons of plastics accumulate in the environment annually due to their stability, poor recycling and low circular use, and they represent an ecological threat to nature and human health. At least 350–400 million tons of plastics are being produced annually worldwide [70], while its production keeps increasing by an average of 7% per annum. In 2014, for example, 311 million tons were produced, among these, 59 million tons in Europe. About 64% of the total European plastic demand is concentrated in five countries: Germany, Italy, France, the United Kingdom and Spain. The main types of plastics are polyethylene, polypropylene, polyvinyl chloride and polystyrene [71]. In 2014, the plastic waste in Europe amounted to 25.8 million tons that were recycled (30%), subjected to incineration for energy recovery (40%) or sent to landfill (30%). Efficient recycling and incineration are only practiced in a few countries, while in most countries, more than 50% of plastic waste is landfilled [71]. The latter undergo photo oxidation, degradation and mechanical disruption that often give rise to small fragments, i.e. microplastic particles [72]. Microplastics, plasticizers and plastic additives as well as added monomers and oligomers can enter surface and/or ground water and accumulate in the environment exerting toxic effects [73]. Approximately 5 to 13 million tons of plastic waste enters the Earth’s oceans every year, as the intentional or unintentional final destination [74]. When this waste is ingested by marine animals, it accumulates in the food chain [72]. The most abundant type of primary microplastics in water environments are fibres [75] that originate from washing synthetic textiles. Replacing synthetic textiles with mycelium-based textiles can be a means to greatly reduce plastic pollution. In addition, fungal materials could replace plastics in a variety of other applications (see above).

Current plasticizers are used, among other things, to improve the workability of concrete, the construction material most used in the world. Plasticizers enable the use of less water by keeping the same viscosity of the concrete, thereby producing a highly workable material that hardens into a strong final product. Plasticizers are typically not covalently bound to the polymers, which, over time, may lead to environmental contamination via leaching [73]. A bio-based alternative to the current fossil-based concrete plasticisers would be lignin functionalized to a higher solubility by the action of fungal or bacterial laccases or other oxidative enzymes. The very hydrophobic lignin is insoluble in water and alcohol but soluble in alkaline solutions. Fungal laccases are prevalent in asco- and basidiomycetes and are typically most active at low pH values of 3–5.5, which is a drawback for their use in functionalizing lignin. Accordingly, laccases have to be identified or engineered that have a shifted optimum pH towards an alkaline pH. One recent successful approach at AB Enzymes has been the engineering of a laccase from the ascomycete *Melanocarpus* *albomyces* using a directed evolution approach, which has led to threefold improved kinetics at pH 9.8 [76]. Notably, the principle applicability of laccase-functionalized lignin has been shown, but immediate commercial implementation is hindered by the general availability and inexpensive cost of fossil-based plasticizers.

To date, very little is known about the biodegradability of petroleum-derived polymers and plastics with fungi and only a few reports are available in the literature. Many basidiomycetes are generally known for their ability to degrade polycyclic aromatic hydrocarbons and are applied in bioremediation processes of contaminated soils and liquids [77–80]. So far, plastic-degrading fungi have been successfully isolated from weathered plastic waste obtained from marine habitats and terrestrial waste treatment facilities. The main fungal genera that were isolated and used for lab-scale plastic degradation, primarily of PET and polyurethane so far are: *Acremonium*, *Alternaria*, *Aspergillus*, *Aureobasidium*, *Cladosporium*, *Debaryomyces*, *Emericella*, *Fusarium*, *Gliocladium*, *Mucor*, *Nectria*, *Neonectria*, *Penicillium*, *Phoma, Plectosphaerella*, *Rhizopus* and *Trichoderma* [81–84]. In addition, enzymes produced by basidiomycetes (*Agrocybe aegerita, Auricularia auricular*-*judae, Bjerkandera adusta, Nematoloma frowardii, Pycnoporus cinnabarinus, Stropharia rugosoannulata*) have been identified and used for plastic degradation. Among the most promising enzymes used for PET and polyurethane treatment have been esterases, cutinases, lipases and lignin-modifying and unspecific macromolecule-depolymerising and hydrolytic enzymes, such as different peroxidases, laccases, glucose oxidases and cytochromes P450. However, no distinct degradation effects for virgin and pre-treated plastics have been achieved solely by the use of pure enzymes [70]. Hence, currently ongoing research is focused on mycelial-based degradation (whole cell degradation) of pure or mixed cultures in combination with purified enzyme cocktails.

### The Janus face of filamentous fungi

The fungal kingdom is huge: six million species are estimated to exist on Earth [85], of which only a small proportion is known. Most are saprophytes and feed on dead plants and animals, and only a few dozen are exploited in biotechnology as cell factories. Leveraging their metabolic capacities and flexibilities will be key to achieving the circular economy outlined above (Fig. 2).

However, many filamentous fungi pose a threat to plant, animal and human life. As comprehensively covered in the first white paper of the EUROFUNG consortium published in 2016 [4], a multitude of plant-pathogenic fungi destroy staple crops annually, which would be enough to feed 600 million people [86]. Human-pathogenic fungi compromise the life of 1.2 billion people worldwide and kill about 1.5–2 million annually, exceeding the fatalities caused by malaria or tuberculosis [87]. Regarding more information on the huge impact of fungal pathogens on human health and food security and the related challenges, the interested reader is referred to the EUROFUNGs white paper from 2016 [4], a review covering the top 10 most feared fungi published in 2018 [88] and to the One Health report published by the American Academy of Microbiology in 2019 [89].

**Fig. 7:**
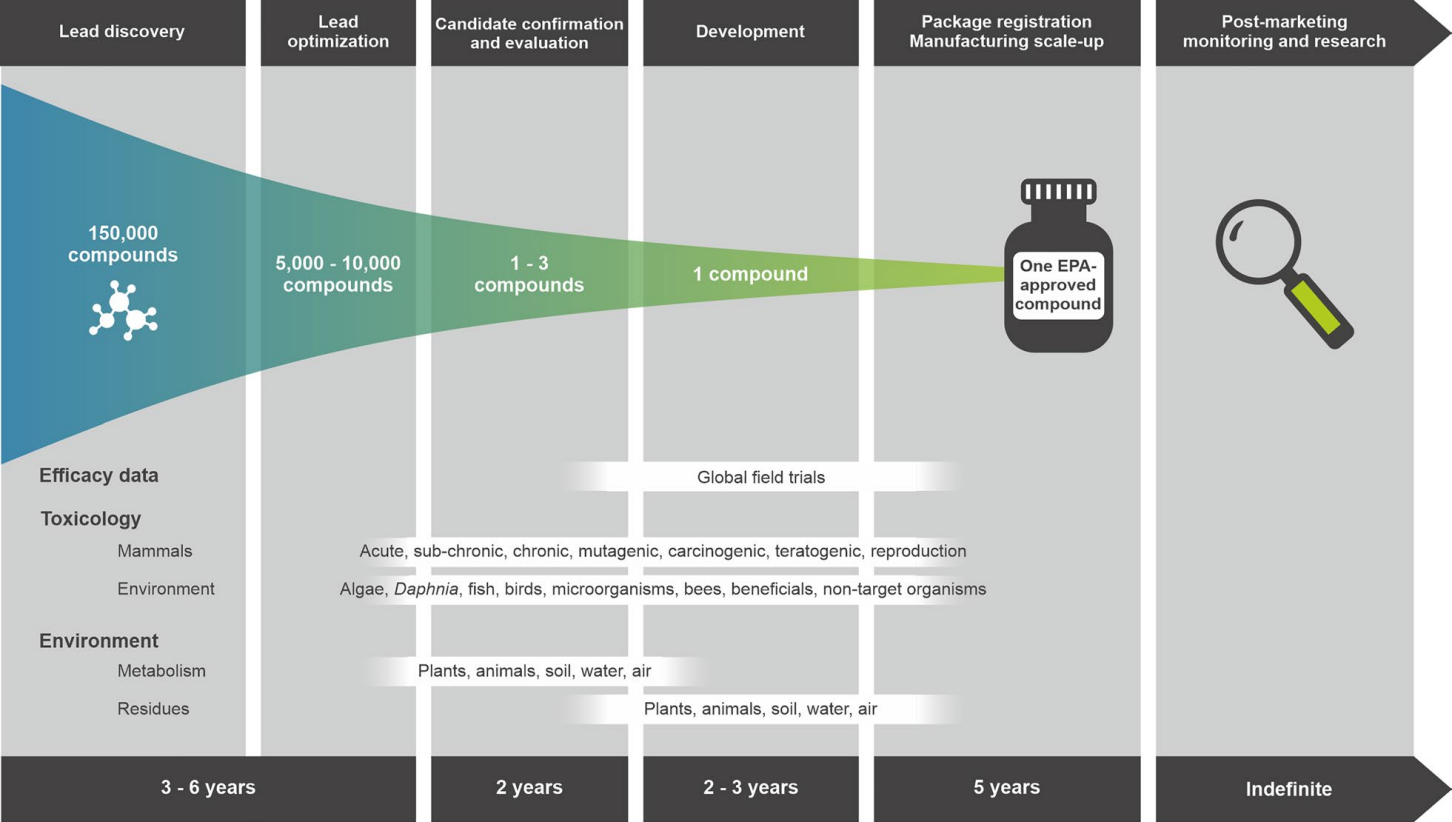
From science to market—the fungicide discovery and approval pipeline. Historically, the biological activity profile (fungicidal potency and spectrum) was used to filter hit compounds to lead molecules. Nowadays, regulatory requirements regarding human and ecotoxicological safety mean that proxy assays for these regulatory requirements are now utilised as early in the discovery pipeline as possible as part of the selection criteria for compound progression. It will typically take 10–12 years for a new fungicide to pass through the various stages of research (lead generation, early and late lead evaluation, optimisation and candidate confirmation) before promotion into evaluation and, finally, product development. After reaching the market, a significant amount of investment is still required for product lifecycle management (e.g. product monitoring feeds into improvements in formulation)

Meeting the agricultural production required to feed the increasing world population [90] is likely to be made more difficult by climate change. This problem is exacerbated by a wide range of plant diseases caused by the diverse group of fungal and newly emerging pathogens. A list of the ten most important fungal pathogens of plants published in 2012 [91] is outdated. If this list had been collated more recently it would have undoubtedly also included the basidiomycete *Phakopsora pachyrhizi*, which causes Asian soybean rust and threatens the sustainability of the millions of hectares of soybean grown in Brazil each year, despite only being detected in Brazil for the first time in 2001 [92]. Soybean production is now only viable in Brazil because of the availability of effective fungal control agents. However, even with the solutions available from the agricultural industry, crop losses remain high in soybean and a wide range of other crops [93]. In many cases, the losses seen are due to fungi that have developed resistance to existing control strategies [94–96]. The speed with which many species of fungi evolve and become resistant to existing control strategies means that there is a constant need to identify agents with novel modes of action. However, the development of novel fungicides by companies active in this field, such as Bayer Crop Science and Syngenta, requires a huge effort and major investment (Fig. 7), taking more than 10 years from the identification of a new screening ‘hit’ to market [97].

The screening of hundreds of thousands of random chemical inputs has historically produced a very low yield of hits, therefore, hypothesis-driven input selection methods are increasingly being utilised. However, the current target assessment and prioritization approaches are still heavily reliant on information from ‘model’ fungal systems, such as *S. cerevisiae* and *Neurospora crassa*, rather than on good quality data produced from fungal pathogens that result in the highest crop losses. It is remarkable that almost all plant pathogens diverged from the fungal ‘model’ systems millions of years ago and have very diverse modes of pathogenicity [98–100]. Therefore, the reliance on these classic ‘model’ systems is deeply unsatisfactory and a significantly better understanding of the genetics and metabolism of fungal plant pathogens is likely to help deliver novel fungal control strategies. In this respect, it is worth mentioning that the EUROFUNGs white paper from 2016 stressed that the term ‘model’ is misleading and advocated a shift in focus towards ‘reference’ strains [4].

Some ubiquitous filamentous fungi which are not pathogenic and usually thrive on dead plant material are still dangerous for human health when consuming crop-based products. Filamentous fungi produce a plethora of so-called secondary metabolites (SMs), of which a subset, the mycotoxins, are harmful. These can be produced by fungi growing either on the crops, some without disturbing plant growth, or post-harvest. The SMs are synthesised by accessory biosynthetic pathways thought to be important for a range of processes, including spore development, spore survival and fungal communication. Recent advances promote the view that fungal mycotoxins are fitness factors that help to establish a niche for protection from competing microbes [101, 102].

Mycotoxins are toxic, carcinogenic and/or mutagenic fungal SMs and can be present in high concentrations in food and feed products. Mycotoxins have significant economic effects on a wide number of crops, most commonly but not exclusively contaminating grain crops (maize, wheat, barley), peanuts and tree nuts, beets, grapes and coffee. The Food and Agriculture Organization has estimated that 25% of the world’s crops are contaminated with unacceptable levels of mycotoxins. Economic losses are not easily quantified, but based on the reports that exist, losses are staggering. Aflatoxin, for example, is the most problematic mycotoxin, with estimated annual losses due to contamination ranging from $52 million to $1.68 billion regarding the US corn crop alone [103]. A relatively small number of ascomycete genera—*Aspergillus, Penicillium, Fusarium, Claviceps* and *Alternaria*—produce most of the common mycotoxins. Major mycotoxins include aflatoxins, citrinin, cyclopiazonic acid, ergot alkaloids, fumonisins, ochratoxin, patulin, trichothecenes and zearalenone [104]. All these metabolites have adverse effects on vertebrate cells depending on the amount ingested and the age and sex of the consumer. Aflatoxin B_1_ is the mycotoxin most studied and the mechanism by which it causes liver cancer is well understood [105]. Trichothecenes, which interfere with protein synthesis in animals and humans, are common contaminates of wheat and a huge problem across all wheat-growing regions in the world. Patulin is of especial concern as it is frequently found in apples and products derived from apples that are popular children’s foods, such as apple sauce and juice [104]. It has also been proposed that SMs cause adverse human health effects after exposure by inhalation (e.g. exposure to moulds indoors; [106]).

Although the molecular genetics of many mycotoxins is well understood [104], efforts to control the contamination of food and feed with these small molecules is still to be realised. Aside from the challenges of controlling mycotoxin contamination using traditional efforts, such as breeding plants for resistance and/or detoxifying contaminated products, several emerging issues have complicated the ability to tackle the mycotoxin problem. Four significant issues to be considered in relation to mycotoxin amelioration are: (i) masked mycotoxins, (ii) climate change, (iii) emerging mycotoxins and (iv) co-occurrence of mycotoxins. Masked mycotoxins refer to plant derivatives of fungal mycotoxins. There is concern and data to support that these hybrid molecules can contribute to toxicity either directly or indirectly through the release of the parent mycotoxin in animal digestive tracks [107]. Research groups worldwide are now examining how climate change might impact mycotoxin synthesis for diverse fungi and crops. Several studies suggest increases in mycotoxin contamination although predictive modelling approaches need to be refined [108–110]. Emerging mycotoxins refers to toxic molecules that occur frequently in products but are not consistently monitored (e.g. eniatins, alternariol, emodin and sterigmatocystin; [111]). The frequency and occurrence of these less common mycotoxins should ideally be subject to risk assessment. Finally, many mycotoxins co-occur in the same crop, yet, worldwide regulations only consider the toxicology of a single mycotoxin [112]. Little is known whether combinations of mycotoxins affect the consumer in an antagonistic, additive or synergistic manner. Despite the wealth of published works on the topic of mycotoxins, substantial gaps remain in the knowledge of how to reduce contamination, what quantities are important in health, whether co-occurrence is a topic to consider and how climate change will impact future risks of these fungal metabolites.

### Measures to advance science on filamentous fungi

Exploiting and fighting fungi for a sustainable, resource-saving bio-economy that operates in a circular manner demands considerable improvements in research and development on scientific and community levels:

- Science: (i) improved understanding of fungal biology in a diverse set of species and strains, including lab (reference) strains, industrially exploited strains and ecologically relevant species; (ii) improved high-throughput technologies and tools to study the dynamics of fungal growth, product formation and pathogenicity and their adaptation to changing environmental conditions; (iii) investment in the standardisation of measures, methods and automation tools; and (iv) investment in open access infrastructures for the storage, analysis and reuse of systems biology data, including biofoundries.
- Community: (i) overcoming fragmentation of the research community studying filamentous fungi; (ii) increasing the number of scientists devoting their lifetime to the study of fungi; (iii) the improved intersectoral and interdisciplinary education and training at undergraduate and graduate levels; and (iv) the engagement of scientists and educators in public outreach and the dissemination and engagement of the general public in fungal science.

The science on filamentous fungi has always been lagging behind that on many other microorganisms, such as yeast and bacteria. Four main reasons for this delay can be specified: firstly, genetic transformation of filamentous fungi is less efficient and more time-consuming compared to unicellular bacteria and yeast [113]. Secondly, genome sequences of filamentous fungi only became available a decade after the genomes of the ‘model’ unicellular fungus *S. cerevisiae* (1996; [114]) and the ‘model’ bacterium *E. coli* (1997; [115]) were released to the public. Thirdly, the genomes of filamentous fungi contain far more genes. A filamentous fungal genome usually carries between 9000 and 14,000 genes, whereas *E. coli* has about 4000 and *S. cerevisiae* about 6000 genes. Finally, there are a few million fungal species, consequently, the research communities studying a particular filamentous fungus are considerably small and fragmented. Each filamentous fungus is an individual of its own, different from all the other fungi, although its outlook is covered by the universal filamentous form. One of the largest of these scientific communities, studying *A. niger*, consists of only about 30 research labs worldwide [2], whereas more than 1800 research labs studying *S. cerevisiae* are registered at the Saccharomyces Genome Database [116].

Some substantial measures for basic and applied science on filamentous fungi are highlighted in the following. These need to be addressed in the short- to mid-term in order to fully leverage the power of these microorganisms for a circular economy.

#### Community engagement and sustainable infrastructures for high-quality fungal omics data

Biology has entered the digital age in many ways, most obviously with the rapid growth of large datasets, including genomes, transcriptomes, proteomes, phenomes and metabolomes. The exploitation of these data underpins the future of biology but requires effective infrastructure, supported and facilitated by easily accessible online technologies delivering high-value analyses. However, this revolution presents major technological challenges and resource implications. This is particularly stark for the fungal research community, due to the diversity of both the organisms and fields of research. On top of this, a vast amount of biological information and data is not in a searchable, digital form but is buried throughout our rich and varied literature.

Omics data is very cost-effective, but its utility is largely dependent on making the data accessible and usable for the wider research community. Every effort should be made to develop the future of genomics-driven fungal research in order that everyone can have full access to reliable genomic information. Consequently, accessing and representing diverse aspects of biology, ranging from phenotypes and subcellular localisation to protein interactions or the geographical location of isolates, in a searchable format is not easily achieved or readily automated. Therefore, a lot of the work required is time-consuming and requires dedicated personnel with biological expertise and information technology skills to apply appropriate classifications and ontologies. New solutions are needed for a large amount of multispecies omics data comparison. Addressing these issues requires co-ordination between researchers, publishers and databases, so that, at the time of publication, key information is deposited in a logical way within these resources. This will require the community to act proactively to facilitate the integration of data, consistency in the use of terminology and gene names, and journals to consider these issues when defining their requirements for data deposition and developing practices to facilitate text mining [117].

Fungal genome sequences are hosted at several fungal databases (e.g. FungiDB [118], JGI MycoCosm [119], Ensembl Fungi [120]), many more genomes are deposited in the NCBI, ENA or DDBJ, and some are hosted by private resources. Free, open access to data is generally accepted as a key priority, which, consequently, requires significant long-term funding for well maintained and resourced, actively developing databases to serve their user communities. Many funding organisations have contributed to setting up and maintaining bioinformatic resources, but the required long-term commitment is difficult to obtain, and with an inevitable cliff edge each time renewal of funding is sought, the reality is that key resources have been lost and will be lost in the future. A consequence of the funding issues is a general drive to centralise, with a number of organism-specific resources having been closed down or frozen (e.g. AspGD), due to a loss of funding. How to address this global issue is not easy and needs both engagement and commitment from funding agencies and/or governments, alongside concerted efforts from the community to shape policy. Alternative funding models must also be considered. However, most proposals potentially endanger the ethos of full open access.

Early genome sequences were limited in quality due mainly to the sequencing technologies used; more recent higher quality and better assembled genome sequences suffer from poor annotation. In the early days, consortia were formed to improve upon the automated gene calling and annotation by manual curation and verification of genes. Nowadays, very few genomes make it past the initial draft annotation [4]. Consequently, errors are likely to occur, and these will be perpetuated and propagated in the annotation of other genomes, influencing downstream experimental analysis. Additionally, comparison of outputs from different bioinformatic pipelines using the same datasets will generally not give exactly the same interpretation—the outputs represent probabilities and models are, thus, not absolute. The resulting differences inevitably complicate the comparison of genomes and other types of data. Lack of awareness or technical understanding can also lead to the misinterpretation and misinformation of any programme or bioinformatic output. This underlines the need for improved training and development, not only for dedicated bioinformaticians but even more for biologists in general. Currently, there is a serious lack of qualified personnel with adequate formal training. Consequently, as a minimum, there is a need for appropriate training regarding accepted rules for omics data handling, enabling fungal biologists to efficiently contribute their domain-specific expertise using ontologies, terminologies and platforms that minimise the need for professional curation and maximise value to the broader user community.

Databases, such as FungiDB and MycoCosm, have a challenge in choosing the organisms and datasets they should host and on where the particular reference genomes, their limited annotation and curation efforts should focus. Consequently, the active engagement with and involvement of researchers is essential in making these decisions. Most researchers inevitably regard their own work as a priority and there is a danger that those with the ‘sharpest elbows’ will prevail. Therefore, it is important that communities ensure they are well represented and informed of decisions, proposed priorities and opportunities.

#### Opportunities and challenges in getting value from fungal omics data

The integration of databases provides opportunities for shared resources and infrastructures which have inevitable cost-savings. ClinEpiDB [121, 122], for example, accommodates a diverse range of large-scale epidemiological datasets for enteric, respiratory and parasitic pathogens but no fungal data. The common infrastructure used by ClinEpiDB and FungiDB could potentially facilitate the integration of epidemiological datasets for fungal pathogens and, as such, allow room for innovation. These resources, allowing the mapping of disease and genomic data, could be potentially developed more broadly for fungal researchers interested in plant disease, ecology and the migration of key pathogens or drug resistance. Additionally, this may facilitate new developments to include the characterisation of transcriptional changes associated with host pathogen interactions [123] or between microbiomes [124].

A major challenge for both the database providers and their users is that data is not static nor is the interpretation of data generally limited to a single definitive result. Genomes, for example, may be re-sequenced using new technologies and additional datasets, such as proteomics or RNA-Seq, are constantly being added. These changes and additions, alongside modifications to bioinformatic pipelines, will facilitate improved gene calling and annotation. Consequently, gene models may be removed or split, misannotated open reading frames revised, differential splicing revealed and untranslated regions, defining the transcription start sites and mature 3′ ends, clarified. These changes can result in difficulties. It is challenging, for example, to map all *N. crassa* knockout mutants to the current gene models hosted by different databases. Researchers, therefore, need to be aware that genomes are not set in stone but evolve with data and technical advances and the databases must make these changes clear and traceable. Updating repository records is paramount to ensure that all databases have access to the most recent information and, as such, provide the best possible service to their users.

Another confounding factor when harnessing genome data of filamentous fungi is the fact that between 40 and 50% of the genes in a filamentous fungal genome are ‘hypothetical’ and lack functional predictions [4]. In addition, only 2–10% of the genes with predicted functions have been experimentally studied in filamentous fungi and, thus, have a verified function [125, 126]. This renders *in silico* reconstructed genome-wide metabolic models considerably incomplete, i.e. with gaps, dead-end reactions and dead-end metabolites. Even for the ‘model’ yeast *S. cerevisiae*, 21% of its predicted genes (i.e. about 1400 genes) still have dubious functional predictions in 2019, which is 22 years after the release of its genome sequence and despite a research community with more 1800 labs worldwide [127]. One powerful approach to overcome this limitation is the interrogation of gene expression networks based on hundreds of transcriptomic datasets available for filamentous fungi. This wealth of data has recently been harnessed to improve gene annotations and assign gene function predictions to 65% of genes predicted in the *A. niger* genome [128], which now can be accessed at FungiDB.

Notably, ecologically important pathogens and endophytic fungi have traditionally received very little support due to the absence of dedicated funding. Those managing the bioinformatics resource need to work with the researchers to define the types of data and criteria that apply when selecting datasets for inclusion so that the process is rigorous, not biased or arbitrary, and that appropriate computational workflows are developed in a timely way. This will also help the researchers to consider relevant criteria when designing their experiments and specific initiatives may be developed to address particular problems. A key issue in confirming gene models, for example, is identification of the 5’ and 3’ ends of transcripts. Most RNA-Seq approaches are poor at defining transcript ends, but specific approaches can be applied that address this directly. In this way, the structural annotation of key genomes could be significantly improved for the wider community. Awareness of such issues may help the research community optimise the utility of their data.

Other on-line resources provide bioinformatic pipelines to assist and minimise the expertise required. One example is Companion [129], which provides a genome production pipeline. The most recent version has proved effective in the production of usable fungal genomes based on the *de novo* analysis of 25 previously characterised reference genomes. The future development and continued support of this and other such initiatives should help the lab-based researchers complete a good first draft of a genome annotation efficiently without major resource implications. Furthermore, recent developments in the Apollo pipeline [130], integrated within databases such as VEuPathDB [131], allows researchers to inspect and modify genome features efficiently and can also be utilised in private workspaces. Data can remain private to a specific work group and be transferred to public databases when appropriate. Consequently, the research community can fully engage to develop shared resources. As these contributions can be attributed to their author, this can be acknowledged by the community and potentially lead to micropublications, incentivising participation and rewarding those who contribute.

Last but not least, the rapid evolution of sequencing and other high-throughput technologies requires active discussions to improve policies and infrastructure for data sharing, integration and analysis in order to resolve the bottlenecks and challenges faced. The changes will not happen overnight, but effective communication should involve all parties concerned, including the on-line database providers, researchers, funding agencies and policymakers. In order for these amazing resources to be used to their full potential for high impact research, these different parties must actively strive to ensure we have effective data sharing policies that will ultimately result in long-term sustainability, accessibility and utility.

#### Improved tools and technologies to study fungal biology

The molecular and analytical toolkit available for filamentous fungi has improved substantially during the 4 years since the first white paper was published in 2016 [4], demonstrating the high innovation potential of the filamentous fungal research community. Genome editing via CRISPR has become routine and is nowadays run in a multiplex manner for many reference strains [132, 133]. Fourier-transformed infrared spectroscopy has become a next-generation phenotyping technology to identify fungal strains and their metabolic products [134], while the power of surface analysis tools, such as mass spectrometry-based imaging techniques, has been exploited to identify changes that are brought about by filamentous fungi and their enzymes when they attack and modify insoluble lignocellulose materials [135]. “Ready-to-use-microfluidics” have been implemented to study the dynamics of fungal growth and fungal interaction with soil bacteria [136], X-ray microcomputed tomography has been successfully applied to study spatial distribution of hyphae within mycelia and diffuse mass transport therein [137, 138], and gene and protein networks have been developed to assist our understanding of filamentous fungal biology holistically [128, 139].

Hence, the technological foundation is sound and powerful and no longer represents a critical hurdle for gaining knowledge on fungal biology. As of now, high-throughput methods for automated cloning, cultivation, mining and screening of fungal strains are, unfortunately, not yet state-of-the-art.

#### Standardisation as a driver for engineering fungal biology

Standardisation is a key for success. Standard reference methods, measures, substrates and materials ensure reliable fabrication and reproducible production characteristic of advanced global industries, such as the semiconductor and forest products industries [140–142]. Engineering biology, broadly called synthetic biology, is similarly benefitting from standardisation [143, 144]. The mycelium materials research community and industry are already benefitting from ASTM and ISO standards developed for products in the forest, textile and acoustic absorbance industries. Accelerating the development and adoption of standard reference materials, measures and methods across the mycelial materials community and beyond could enable advancements in basic science on filamentous fungi and engineering of fungal-based bioproduction analogous to the advancements in the semiconductor industry. New options for sharing mycelium materials that reduce transaction costs, allow for redistribution and enable commercial production could provide the incentive for the broad adoption of standard references, measures and methods that support reliable fabrication and reproducible science [145, 146].

Reliable, standardised measurements are critical to reproducible science [147]. Metrology formalizes reliable measurements, and advances in metrology for biological systems are greatly benefitting basic science and the engineering of biology [148, 149]. Characterising organisms, substrates and production environments in real-time across orders of magnitude in the scale of space and time could accelerate the development of advanced fungal products while, simultaneously, benefitting basic science and the engineering of biological systems. As an example, standard metrological materials, such as well-characterised 1 cm^3^ units of mycelium material, and metrological methods could help to co-ordinate practitioners across locations and resource settings, reduce manufacturing costs, reduce time to market and disseminate best practices [150].

The confluence of standard approaches and automation tools characteristic of modern semiconductor foundries have cultivated the semiconductor industry into one of the most significant in human history. Recent advancements in the automated construction of synthetic organisms are making biofoundries economically viable and they are increasingly being recognized by the life sciences research community as an essential infrastructure for basic and applied research [151, 152]. Companies such as Gingko Bioworks and Ecovative Design have invested substantial capital in biofoundries for constructing organisms and there is an emerging ecosystem of “cloud laboratories” that aim to provide practitioners access to automated labs without the need for large capital investments [153–156]. Biofoundries with capacities for constructing filamentous fungal organisms could build on recent work to synthesize the genomes of the bacteria *E. coli, Caulobacter crescentus* and the yeast *S. cerevisiae*, with the ultimate goal of enabling advancements in synthetic filamentous fungal organism construction analogous to the construction of JCVI-sc3.0 and Sc2.0 [157–160]. Work to insulate biological systems from evolutionary drift can enable the deployment of engineered biology for applications outside the research laboratory or commercial bioreactors, while mitigating biosafety and biosecurity risks [161]. Such is the promise of distributed fungal-based bioproduction that biofoundries, for example, for mycelium materials in support of basic science, engineering and production, strategically placed across the planet, could ramify into impacts surpassing that of the semiconductor industry.

## Concluding remarks

**Fig. 8:**
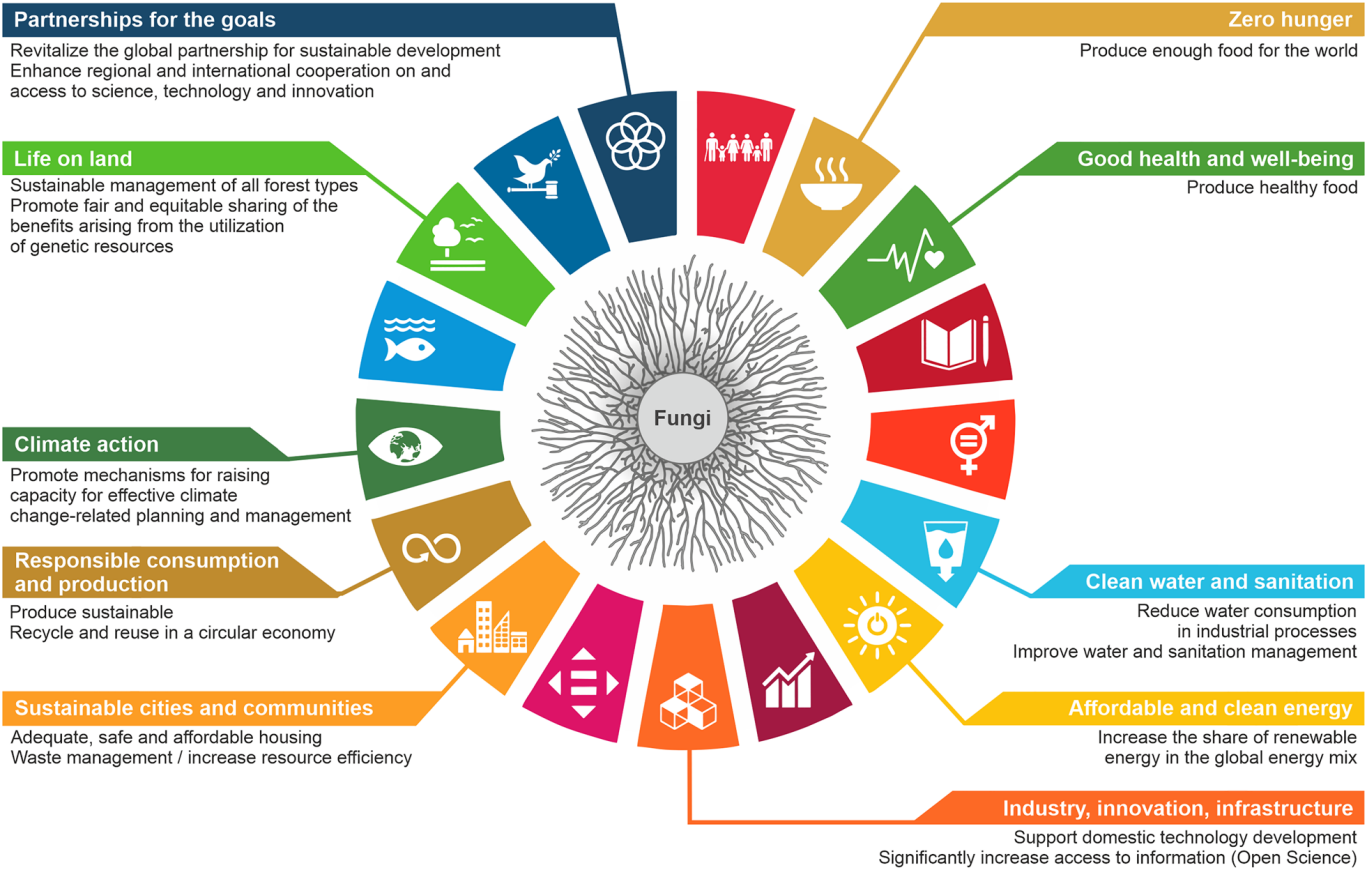
Fungal biotechnology has the potential to make a significant contribution meeting 10 out of 17 United Nation’s sustainable development goals through the rational improvement of filamentous fungal cell factories

We would not be able to live the life we are living without the help of moulds and mushrooms from nature. Fungi are our present and they will shape our future. They are champions in recycling and material transformation; their biosynthetic capacities are unmatched in the microbial world. We should do our best to harness their abilities! Fundamental and applied science on fungi offers solutions for the shift from our current petroleum-based economy into a bio-based circular economy, opens new avenues for food security as demand increases from a growing human population, and provides us with new concepts on how to ensure human, animal and plant health in the future. Science on fungi discussed in this white paper already contributes to 10 out of 17 UN development goals (Fig. 8) and their role will become even more important for the future of mankind.

The economist Peter Drucker (1909–2005) once said: “The best way to predict your future is to create it.” Stronger mutual collaborations between scientists, engineers, artists, designers and industrial stakeholders, and vivid communication with the general public and policymakers will ensure that the inter- and transdisciplinary science on fungi will create a path towards innovative breakthroughs. As Peter Drucker suggests, this will create a sustainable economic future based on fungal cell factories for years to come.

### Supplementary information

**Additional file 1: Table S1.** Protein digestibility corrected amino acid (PDCAA) scores.

## Data Availability

Not applicable.

## Appendix

See Table 3.

**Table 3:** Participants of the meeting

Arnold Driessen	University of Groningen
Arthur Ram	Leiden University
Bernhard Seiboth	Technische Universität Wien
Bertram Schmidt	Technische Universität Berlin
Carsten Pohl	Technische Universität Berlin
Charlotte Steiniger	Technische Universität Berlin
Charlie Cairns	Technische Universität Berlin
Christian de Lutz	Art Laboratory Berlin
David Canovas	University of Seville
Derek Carr	Kerry Group
Eric Record	French National Institute for Agriculture, Food and the Environment
Erzébet Fekete	University of Debrecen
Gerhard Braus	University of Göttingen
Guliano Sciara	French National Institute for Agriculture, Food and the Environment
Han Wösten	Utrecht University
Hans van den Brink	Chr. Hansen A/S
Istvan Posci	University of Debrecen
Jens Frisvad	Technical University of Denmark
Jolanda van Munster	University of Manchester
Kristiina Hilden	University of Helsinki
Levente Karaffa	University of Debrecen
Marja Paloheimo	Roal Oy
Mark Caddick	University of Liverpool
Miia Mäkelä	University of Helsinki
Michael Csukai	Syngenta
Nada Kraševec	National Institute of Chemistry Slovenia
Nancy Keller	University of Wisconsin-Madison
Nina Gunde-Cimerman	University of Ljubljana
Nefertiti Campos	Puratos
Oded Yarden	Hebrew University of Jerusalem
Peter Punt	DutchDNA
Philip Ross	MycoWorks
Philipp Benz	Technical University of Munich
Rasmus Frandsen	Technical University of Denmark
Regine Rapp	Art Laboratory Berlin
Rob Johnson	Quorn Foods
Rolando Perez	Stanford University
Ronald de Vries	Westerdijk Fungal Biodiversity Institute
Stefan Haefner	BASF SE
Tabea Schütze	Technische Universität Berlin
Thomas Haarmann	AB Enzymes
Uffe Mortensen	Technical University of Denmark
Vera Meyer	Technische Universität Berlin
Volha Shapaval	Norwegian University of Life Sciences
Wolfgang Hinterdobler	Austrian Institute of Technology
Yitzhak Hadar	Hebrew University of Jerusalem
