# Supplementary material for: Growing a circular economy with fungal biotechnology: a white paper
Source: Fungal Biol Biotechnol. 2020 Apr 2;7:5. doi: 10.1186/s40694-020-00095-z (PMC7140391; doi:10.1186/s40694-020-00095-z)
Supplement: Supplementary file 1 — Additional file 1: Table S1. Protein digestibility corrected amino acid (PDCAA) scores. [file 40694_2020_95_MOESM1_ESM.docx]

**Table S1.** Protein Digestibility Corrected Amino Acid (PDCAA) Scores.

| (PDCAAS) of Selected Food Proteins | | |
| --- | --- | --- |
| Protein Source | PDCAAS | Data Source |
| Quorn pieces | 1.0 | (iv) |
| Casein | 1.0 | (i) |
| Egg white | 1.0 | (i) |
| Chicken (light meat-roasted) | 1.0 | (iii) |
| Mycoprotein | 0.99 | (iv) |
| Turkey (ground-cooked) | 0.97 | (iii) |
| Fish (cod – dry cooked) | 0.96 | (iii) |
| Soybean protein | 0.94 | (ii) |
| Beef | 0.92 | (i) |
| Pea flour | 0.69 | (i) |
| Kidney beans (canned) | 0.68 | (i) |
| Rolled oats | 0.57 | (i) |
| Lentils (canned) | 0.52 | (i) |
| Peanut meal | 0.52 | (i) |
| Whole wheat | 0.40 | (i) |
| Wheat gluten | 0.25 | (i) |

Sources: (i) FAO/WHO Joint Report 1989, ISBN 978-92-5-107417-6; (ii) Sarwar and McDonough, 1990, PMID:

2198245. (iii) Calculated from amino acid data in USDA Nutrient Data Base for Standard Reference, March 12, 1998 (assumes a digestibility equivalent to beef = 94 %). (iv) Calculated from Marlow Foods data.
